# Business Owner-Managers’ Job Autonomy and Job Satisfaction: Up, Down or No Change?

**DOI:** 10.3389/fpsyg.2020.01506

**Published:** 2020-07-10

**Authors:** Sukanlaya Sawang, Peter Joseph O’Connor, Robbert A. Kivits, Paul Jones

**Affiliations:** ^1^Faculty of Business and Law, Coventry University, Coventry, United Kingdom; ^2^QUT Business School, Queensland University of Technology, Brisbane, QLD, Australia; ^3^School of Business and Tourism, Southern Cross University, Lismore, NSW, Australia; ^4^School of Management, Swansea University, Swansea, United Kingdom

**Keywords:** job autonomy, longitudinal, job satisfaction, self-employment, small business

## Abstract

The current study developed a dynamic model which identified a pattern of change in small business owner-managers’ job autonomy and job satisfaction separately through the trend analyses (linear, quadratic, and cubic trends). The current study then tested the associations between the growth models of job autonomy and job satisfaction. The study utilized data from an Australian sample over 9 years with a total sample of 1,044 self-employed individuals. In brief, the findings illustrate a curvilinear relationship (cubic and non-monotonic) between changes in job autonomy and job satisfaction. Further, the change rate of job satisfaction was faster among small business owner-managers who perceived greater fluctuation of job autonomy, compared to those who perceived lesser shifts in job autonomy.

## Introduction

Several studies indicate that a primary motive driving business start-up is job autonomy (Shane et al., 1991; Birley and Westhead, 1994; Gatewood et al., 1995; Feldman and Bolino, 2000; Carter et al., 2003; Van Gelderen and Jansen, 2006). Job autonomy is viewed as a degree to which the job provides individuals with freedom, independence, and discretion in work scheduling, decision making, and work methods (Morgeson and Humphrey, 2006). Using panel data from 25 European countries with 11,157 observations, Lange (2012) concluded that job autonomy has a strong and positive impact on the probability of someone being a small business starter^1^. A study using US panel data also showed that transitioning from regular employment to self-employment is associated with increases in job satisfaction (Hundley, 2001). Similarly, a large study of the British populace identified that individuals’ life satisfaction increased for 2 years after changing a career from regular employment into self-employment despite earning less and working longer hours (Binder and Coad, 2013). Collectively, these results indicate that autonomous work – which is a core feature of start-ups – is associated with positive subjective well-being and can serve to counter the negative aspects of start-ups such as increased workload. Unfortunately, Binder and Coad’s study only measured job satisfaction twice over a 2-year interval. With two time points, we can have only a linear assessment of change and the question remains: how do business owner-managers perceive the level of job autonomy over time and how are changes in autonomy associated with changes in subjective well-being?

Only limited research has examined patterns of job autonomy among small business owner-managers. One recent study, however, sampled 61 business owners and found that business owner-managers’ experiences of job autonomy vary over time (Van Gelderen, 2016). They reported that business ownership may start with experiencing high levels of job autonomy, but that this may be reduced at certain points in time due to competing interests with other stakeholders (e.g., suppliers and business partners). However, Van Gelderen’s study did not track the autonomy of business owner-managers over time and consequently did not empirically capture the dynamic patterns of job autonomy or its consequences. Indeed, the importance of studying job autonomy dynamically was highlighted by Van Gelderen (2016), who suggested that “autonomy is best studied over time” (p. 560).

This article uniquely contributes to the small business literature in two important ways. First, the current study employs 9 years of job autonomy data, using latent growth modeling (LGM) to describe and predict patterns of change over time in autonomy. While the psychological adaptation theory (Zapf et al., 1996) and self-determination theory (SDT; Gagné and Deci, 2005) seem to suggest that the job autonomy is not stable and can be changed, the current literature does not provide the pattern of change. For example, job autonomy can be *dropped* to a certain point of time, then *remains* for a period of time before it *dropped* again (drop-remain-drop). It can also be possible that job autonomy can be *dropped* to a certain point of time, then *increased* to a period of time before it *dropped* again (drop-increase-drop). Perhaps job autonomy can be *increased* to a certain point of time, then *remains* for a period of time before it *increased* again (increase-remain-increase). LGM can analyze the starting point of growth (intercept), shape of growth over time (linear or non-linear), and the rate of growth (slope) over time (see the “Data Analysis” section). The current study thus can measure the growth patterns of job autonomy over time and model the shape of growth accordingly (i.e., no growth, linear growth, or curvilinear growth).

Second, the study examines to what extent the rate of growth over time in job autonomy impacts the growth rate in job satisfaction. Drawing from a widely used model of job demand-control (Karasek, 1979), work autonomy can mitigate the negative effectives of high demands on psychophysiological outcomes, which is called the buffer hypothesis. Researchers who have examined this hypothesis have found that increasing job demands have almost no impact on subjective well-being as long as individuals’ job autonomy to make decisions (as a moderator) is also enhanced (Wall et al., 1996; Brough and Biggs, 2015; Dawson et al., 2016; Nguyen and Sawang, 2016; Cendales-Ayala et al., 2017). As businesses grow, job autonomy may fluctuate when business owner-managers deal with a range of stakeholders such as customers, suppliers, competitors, or regulators (Van Gelderen, 2016). The current study aims to empirically examine whether job autonomy changes over time as suggested by Van Gelderen’s qualitative work. Additionally, the current study aims to understand the longer-term effect of job autonomy by examining whether the rate of change in job autonomy impacts change in job satisfaction over time.

### The Role of Job Autonomy

Autonomy is a psychological construct, which refers to the sense of discretion, freedom, and independence, to individuals striving toward the development and realization of personal goals, values, and interests (Assor et al., 2002). Drawing from the demand-control model (DCM; Karasek, 1979), job control, as in the use of skills and job autonomy at work, can assist individuals to cope with work demand. Although the DCM is widely used to explain the demand-control relationship among employees, the model is also relevant in the present context because business owner-managers are also required to deal with their stakeholder demands (so called job autonomy-related tension) and decisional freedom (Van Gelderen, 2016).

Perceived autonomy can be described as an affective experience which becomes a: “*self-generated affective kick when they perform well and this internal reinforcement serves as an incentive for continued good performance*” (Hackman and Oldham, 1975, p. 60). The perception of autonomy is an important buffer of negative stress (Mills et al., 2008; Sawang, 2012). Thus, the current study uses the perception of autonomy at work, which plays a critical role in promoting positive outcomes, such as job satisfaction because job autonomy can be regarded as controllability over ones’ work which then can mitigate the negative effects of stressful job on individuals’ psychological well-being (Karasek, 1979; Hessels et al., 2017; Warr and Inceoglu, 2017). Job autonomy was originally viewed as a level of freedom and independence to carry out individuals’ work assignment (Hackman and Oldham, 1975). The concept of job autonomy has been expanded from the original view by reflecting three interrelated aspects centered on freedom in (a) work scheduling, (b) decision making, and (c) work methods (Morgeson and Humphrey, 2006).

In the entrepreneurship literature, there is a variety of reasons for undertaking a business start-up, but job autonomy is often regarded as a prime reason (Gatewood et al., 1995; Carter et al., 2003). Through the media, the entrepreneurial career is often portrayed as an ideal career which provides greater job autonomy, flexibility, a sense of ownership, and a potential of high earnings than organizational employees (Martins, 2011). Empirical evidence highlights the association between occupation and perceived job autonomy, that is, entrepreneurs are more likely to report higher job autonomy than other professionals (Blanchflower and Oswald, 1998, 2000; Benz and Frey, 2004, 2008a; Blanchflower, 2004; Kawaguchi, 2008). The qualitative interviews of 167 entrepreneurs whom were motivated by job autonomy described the job autonomy as (a) decisional freedom, (b) regulating one’s own time, (c) freedom from boss/(organizational) rules, (d) being in control, and (e) self-endorsement (Van Gelderen and Jansen, 2006).

### Stable or Change Over Time?

Some authors make the assumption that autonomy is a constant in entrepreneurs “Autonomy may not be an issue among independently owned and managed entrepreneurial firms because such founders are already acting autonomously” (Lumpkin et al., 2009, p. 63). Yet, it is debatable whether job autonomy is stable or changes over time. Recently, Van Gelderen (2016) explains how the small business owner-managers’ experience of job autonomy changes over time. Drawing from 61 interviews, the study explained that during the entrepreneurial career, business owner-managers may experience the movement of job autonomy between current experienced job autonomy, temporarily sacrificed job autonomy (e.g., very important assignment or for a very important customer), and involuntary lost job autonomy (e.g., financial constraint). Although the experience of job autonomy may be decreased, business owners-managers are more likely to make an effort to attain and maintain job autonomy (Van Gelderen, 2016).

Extending this perspective by drawing from the psychological adaptation theory lens (Zapf et al., 1996), it is plausible that job autonomy can temporally decline. According to the adaptation theory, “…exposure to earlier stimuli serves as a frame of reference by which later stimuli are judged” (Bowling et al., 2005, p. 1046). Through this lens, job autonomy can be adjusted over time and return to a more positive level, despite whether a situation has been improved or not. This phenomena can be also be explained by SDT, which highlights a link from the central role played by job autonomy in thriving personally (Gagné and Deci, 2005). The theory thus suggests that business owner-managers may try to maintain or regain their job autonomy either to fulfill their intrinsic (challenge in seeking job autonomy) or extrinsic (business growth) motivations. This view suggests a non-linear pattern of job autonomy over time. As suggested by Van Gelderen (2016), business owner-managers can perceive the movement of job autonomy at some points in their career. When job autonomy is at risk, individuals may temporarily compromise their job autonomy for a certain situation. Nonetheless, business owner-managers are more likely to put effort in regaining the control back (Van Gelderen, 2016). Drawing from SDT and Van Gelderen (2016) qualitative study, we propose that the change of job autonomy over time should be a curvilinear model rather than a linear model.

Hypothesis 1: Over time, there will be a curvilinear change in job autonomy among small business owner-managers over time.

### Impact of Autonomy on Job Satisfaction

Compared to job autonomy, studying job satisfaction over time is more common, because behavioral economic researchers are interested in the effect of self-employment career transition and happiness (Frey and Stutzer, 2005; Andersson, 2008; Binder and Coad, 2013; Guerra and Patuelli, 2016). Recent studies using longitudinal data highlight that there is a difference of job satisfaction and life satisfaction level before and after career transition into self-employment (e.g., Binder and Coad, 2013; Georgellis and Yusuf, 2016), such that overall job satisfaction and life satisfaction greatly increases within the first year of career change but significantly declines in subsequent years. The inverted U-shaped pattern of job satisfaction is also reflected in a similar pattern among employees who switch between companies (Boswell et al., 2009). Similarly, a study of the German Socio-Economic Panel during 1984–2009 highlighted that switching into a self-employment career significantly increases job satisfaction and remains for 3 years before it decreases back to the *ex-ante* level (Hanglberger and Merz, 2015). Thus, it can be proposed that the growth pattern of job satisfaction among small business owner-mangers can be described as curvilinear. Prior to considering the next research question (to what extent does the growth rate of job autonomy impacts on the growth rate of job satisfaction?), the current study will examine the growth model of job satisfaction.

Hypothesis 2: Over time, there will be a curvilinear change in job satisfaction among small business owner-managers over time.

Being ones’ own boss can be stressful. In fact, research has posited that small business owner-managers report higher psychological distress than employees (Chay, 1993; Jamal, 1997). This can be explained by a high level of investment in psychological and physiological resources (Dolinsky and Caputo, 2003). This does not mean business owner-managers work more than employees. However, the nature of work requires small business owner-managers to have diverse roles, often work alone, and bear the cost of their mistakes (Cardon and Patel, 2015).

The DCM (Karasek, 1979) explains that job demand (stress sources in the work environment) can increase work-related stress. Some studies demonstrate that business owner-managers experience higher levels of work-related stress than employees (Lewin-Epstein and Yuchtman-Yaar, 1991; Jamal, 1997; Blanchflower, 2004). Nonetheless, some existing studies find business owner-managers perceive lower stress than employees (Rahim, 1996; Baron et al., 2016; Hessels et al., 2017) while others find no significant difference between the two occupational groups (Parslow et al., 2004; Andersson, 2008). Using DCM as a framework, these results indicate that job demand can increase with almost no threat to psychological outcomes (e.g., stress and job satisfaction) as long as individuals can maintain their job autonomy (de Jonge et al., 2010). In addition, the difference in psychological outcomes is not because of changes in level of job demands but the decision authority over one’s job (Hessels et al., 2017).

Although an overwhelming number of empirical studies support the positive impact of job autonomy on job satisfaction (e.g., Cooper and Artz, 1995; Fairbrother and Warn, 2003; Hytti et al., 2013), it is unknown to what extent this relationship changes over time. Would perceived job satisfaction be altered based on the changed pattern in job autonomy? Could it be possible that perceived job satisfaction is continuing to grow over time despite the reduction of job autonomy because small business owner-managers see a challenge in seeking/regaining job autonomy or realize the situation is a temporary sacrifice? (Van Gelderen, 2016).

**Figure 1:**
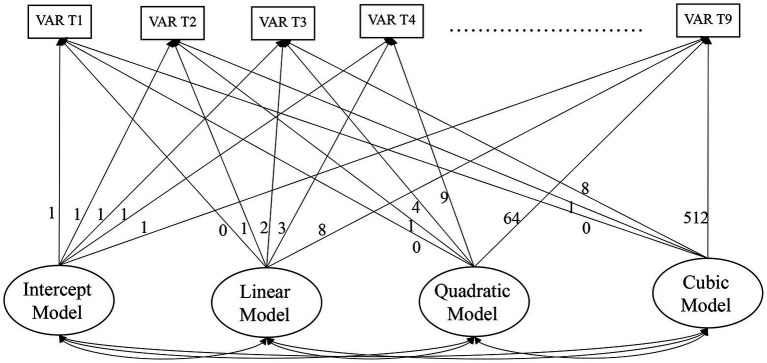
Unconditional latent growth modeling (LGM) models covariates testing linear, quadratic, and cubic UG models as nested models (the autonomy linear model was nested within the autonomy quadratic model; the autonomy quadratic model was nested within the autonomy cubic model-then repeated for job satisfaction) with all possible combinations of fixed and randomly varying slopes to determine which model demonstrated the best model fit.

**Figure 2:**
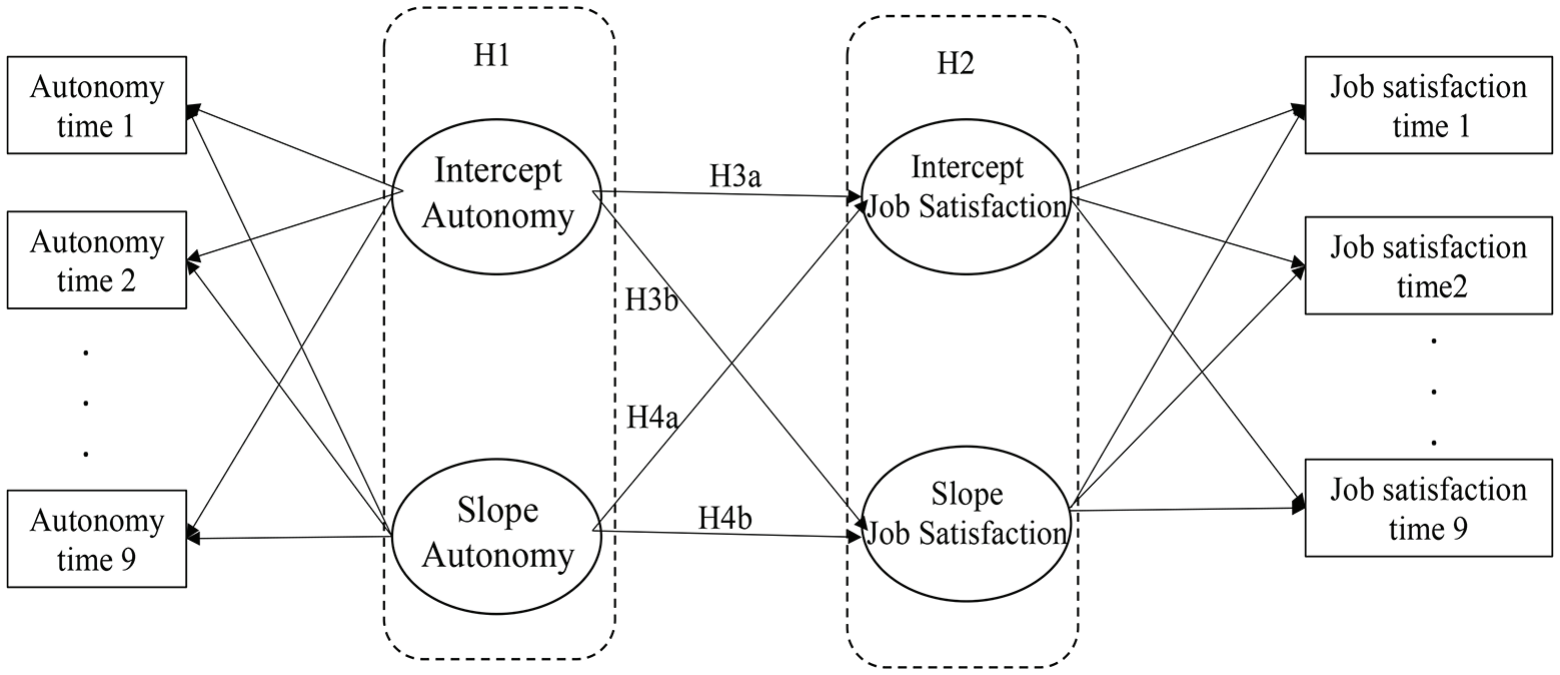
Hypotheses 1 and 2 are tested independently as illustrated in Figure 1. Parallel process model including intercepts and slopes for autonomy and job satisfaction. For conditional model, covariates (gender, age, occupational tenure, business income, number of employees, and solo business owner-managers) paths were added on both intercepts and slopes.

Several empirical studies have examined the impact of job control on psychological outcomes (e.g., de Jonge et al., 2010; Hessels et al., 2017), yet few studies have evaluated this over time [see meta-analytic paper by De Lange et al. (2003)] and most previous studies have used a two-wave design and multiple regression analysis. Moreover, a limited number of studies have explored the role of job autonomy and psychological outcomes over time in self-employment or entrepreneurship context. The recent study by Hessels et al. (2017) examined the DCM between self-employed and employees, using eight-wave longitudinal data. Using pooled Ordinary Least Squares regression, the study found that self-employed participants perceive less work-related stress than employees due to the difference in perceived job control. Despite using multi-wave data, the study was unable to demonstrate a changing pattern of job control over time. The current study extends knowledge on the role of job autonomy by examining whether job satisfaction follows a change pattern of job autonomy, and if so, what this dynamic relationship might look like. As discussed earlier, much literature established the cross-sectional relationship between job autonomy and job satisfaction (Federici, 2013; Rodríguez et al., 2016). Wu et al. (2015), using data with two different time lags, highlighted the positive relationship between job autonomy and job satisfaction among employees. However, the current literature has little to say regarding the growth patterns between job autonomy and job satisfaction, particularly among business owner-managers. Drawing from exiting knowledge that job autonomy positively links to job satisfaction, the current study proposes that change in job autonomy could have a positive influence on the change in job satisfaction over time (slope models). Apart from slope models, the current study also examines the intercept models (initial status latent variable), which closely approximate that used in the cross-sectional (one point in time) designs characterizing the vast majority of prior research. Figures 1, 2 represent the studied models. The intercept and slope models will be further explained in the following section.

Hypothesis 3a: At a giving time, job autonomy (intercept) will increase the predictive effect on job satisfaction (intercept).
Hypothesis 3b: At a giving time, job autonomy (intercept) will increase the predictive effect on increased job satisfaction (slope).
Hypothesis 4a: Over time, increased job autonomy (slope) will have a positive predictive effect on job satisfaction (intercept).
Hypothesis 4b: Over time, increased job autonomy (slope) will have a positive predictive effect on increased job satisfaction (slope).

### Data Analysis

LGM analysis is used to identify the change pattern of job autonomy and job satisfaction. Each business owner-manager may develop the trajectory of job autonomy and job satisfaction differently. LGM allows researchers to capture not only an individual’s developmental trajectory but also captures individual differences in this trajectory over time (Duncan and Duncan, 2009). Thus, LGM can be used to estimate between-person differences in within-person change (referred to as time trends, time paths, growth curves, or latent trajectories). The LGM approach is “*highly flexible in terms of the inclusion of a variety of complexities including partially missing data, unequally spaced time points, non-normally distributed or discretely scaled repeated measures, complex nonlinear or compound-shaped trajectories, time-varying covariates (TVCs), and multivariate growth processes*” (Curran et al., 2010, p. 124). LGM allows researchers to understand the trend of business owner-managers’ perception over time by mapping the developmental patterns over two or more period of times (Preacher et al., 2008).

LGM is an application of structural equation modeling (SEM), and it is used to study the change trajectory of variables by allowing for both latent variables and random coefficients across individual development trajectories. LGM uses maximum likelihood estimation and is able to handle missing data (e.g., participants may drop out from a study) by giving more weight to individuals with the most time points. LGM can calculate starting point of growth (*intercept/initial status*), shape of growth (functional form such as linear or curvilinear), and rate of growth (*slope/rate of change*) over time.

To test Hypotheses 1 and 2, growth trajectories of job autonomy and job satisfaction over time, the current study examined a series of unconditional LGM models as (a) intercept only (ϒ_tj_ = π_0j_ + e_tj_); (b) intercept and linear slopes (ϒ_tj_ = π_0j_ + π_1j_a_tj_ + e_tj_); (c) intercept, linear, and quadratic slopes (ϒ_tj_ = π_0j_ + π_1j_a_tj_ + π_2j_a_tj_ + e_tj_), and (d) intercept, linear, quadratic, and cubic slopes (ϒ_tj_ = π_0j_ + π_1j_a_tj_ + π_2j_a_tj_ + π_3j_a_tj_ + e_tj_). ϒ_tj_ = the observed score at time t for subject j, π_0j_ = constant for subject j, π_1j_a_tj_ = linear growth rate for subject j, π_2j_a_tj_ = quadratic growth rate for subject j, π_3j_a_tj_ = cubic growth rate for subject j, a_tj_ = specified basis term to correspond to the interpretation of the growth factors as the constant (fixed at 1), linear time (t + 1), quadratic (time 1^2^) and cubic trend (time 1^3^), respectively. The graphical representation of testable models can be seen in Figure 1. The measurement model represents individual growth in each construct, with two latent growth parameters (intercept and slope). The Akaike Information Criterion (AIC) and Bayesian Information Criterion (BIC) were used to compare the best fitting model, with lower values indicating better fit (Vasantha and Venkatesan, 2014). Once the growth trajectory of each construct (within-person model) are specified, the parallel process model (between-person model) can be examined.

The next step is to test Hypotheses 3 and 4. The rate of change is the speed at which level of perceived job autonomy and job satisfaction change over a specific period of time. To explore the growth trajectories of job autonomy and job satisfaction simultaneously, the current study examined the parallel process model (Figure 2). This model evaluated two intercepts and slopes in order to explore the impact of change in job autonomy on the change in job satisfaction (H3 and H4).

To understand how rates of change in job autonomy predicted rates of change in job satisfaction over time, the current study examined the parallel process model (Figure 2). This approach is used when trajectories of change or growth processes in two or more variables in parallel (in this case job autonomy and job satisfaction). The parallel process model allows the associations among the growth factors of job autonomy and job satisfaction, in order to examine whether the intercept and growth in one is related to the intercept and growth in the others. “*This (approach) offers a very powerful analytic approach for the study of stability, change, and development across time in multiple psychological variables*” (Wright et al., 2013, p. 3). Finally, controlled variables are added in the conditional model, confirming the relationship between change in job autonomy and job satisfaction.

## Materials and Methods

### Respondents

Data was derived from the Household, Income and Labour Dynamics in Australia (HILDA) Survey, which is a longitudinal, multidisciplinary cohort study on a wide range of factors related to economic and personal well-being, labor market dynamics, and family life (Summerfield et al., 2016). The study utilized the total sample of self-employed individuals who were tracked in the survey across 9 years from 2005 to 2013 (denoted as Time 1 to Time 9) because the job autonomy were assessed in these periods. The interview of each household which was selected from 488 Census Collection Districts in the first wave and followed up with each household in each subsequent wave. Some respondents could drop out in 1 year and come back in the following year. As a result, the response rate of the waves under investigation was 88.62%^2^, and the average dropout among participants for each wave was 39%. The number of self-employed individuals were 901 in Wave 1; 858 in Wave 2; 805 in Wave 3; 769 in Wave 4; 860 in Wave 5; 840 in Wave 6; 1,112 in Wave 7; 1,029 in Wave 8; and 1,018 in Wave 9. The total respondents of all waves were 8,192. However, there were only 1,044 self-employed individuals (13%) completed the data for at least five waves (it is suggested the five waves as minimum to test cubic growth; Rojas and Iglesias, 2013).

Descriptive statistics are provided for the baseline (2005) observation. The sample (*N* = 901) consisted of more men (61%) than women (39%) and their ages ranged from 19 to 86 years^3^ with a mean age of 47. The gender and age characteristics of the current sample are similar to other self-employment studies (Simoes et al., 2016). The average occupational tenure was 15 years. There was an equal split in the number between solo business owner-managers (50%) and non-solo individuals (50%). Of these, seven out of nine non-solo business owner-managers (78%) employ up to four employees. Ninety-five percent of small business owner-mangers reported positive annual income, with an average of AUD 31,825. Five percent reported a negative income, with an average of AUD-15,523. The industry sectors were made as follows: construction (23%), real estate (18%), agriculture (12%), and others^4^.

### Measures

#### Job Autonomy

Six questions^5^ assess (1) work-scheduling autonomy, (2) decision-making autonomy, and (3) work-methods autonomy, rating on a scale of 1 (strongly disagree) to 7 (strongly agree). These questions have been used in numerous other studies measuring job autonomy (DiRenzo et al., 2011; Wu, 2016; Hessels et al., 2017). The average internal reliability is *α* = 0.85 or higher for the entire study period. Higher values mean increased levels of job autonomy. A one-factor congeneric measurement model^6^ revealed factor loadings of each item ranging between 0.61 and 0.94, thus illustrating the evidence of convergent validity. To examine the common method variance (Podsakoff et al., 2012), Harman’s single factor score was used, in which all items (job autonomy and job satisfaction) were loaded into one common factor. The result showed that factor loadings of job satisfaction was poor (0.20) and non-significant. This suggested that the common method variance did not affect the data.

#### Job Satisfaction

A single-item measure of overall job satisfaction was used (Summerfield et al., 2016), rating from 0 (totally dissatisfied) to 10 (totally satisfied). The use of single-item measure is commonly used to eliminate the specifics and the peculiarities of jobs as well as for a different time span (Oshagbemi, 1999).

#### Control Variables

Demographic variables (age, gender, and occupational tenure), industry, firm size in term of employee number and business income, and type of business owner-managers (solo or non-solo) are considered. Gender was included as men perceive on average more overall job autonomy than women (Adler, 1993; Sloane and Williams, 2000). However, women tend to demonstrate greater happiness at work than men (Clark, 1997). Age^7^ and job tenure were also included because older individuals were likely to report higher job autonomy than those who are younger and lower educated (Shields and Price, 2002). The binary variable that distinguishes between solo (no employee) and non-solo business owner-managers was also included. The size of business may also influence the perception of job autonomy as business owner-managers may have to deal with more stakeholders.

## Results

### Unconditional Growth Models

Means, standard deviations, and intercorrelations among the study variables are shown in Table 1. To determine the shape of the change trajectory of job autonomy and job satisfaction, unconditional LGMs (without covariates) were first fit to the data. The significant intercept term indicates the substantial difference in baseline levels of each variable. The significant slope term indicates individual differences in the progression of each variable over time. Fit indices for each model are presented in Table 2.

**Table 1:** Means, standard deviations, and intercorrelations among the study variables.

Job satisfaction										Covariates					
Autonomy	Wave 1	Wave 2	Wave 3	Wave 4	Wave 5	Wave 6	Wave 7	Wave 8	Wave 9	No. of employee	Solo	Tenure	Sex	Age	Income
Wave 1	0.204^**^	0.168^**^	0.128^**^	0.155^**^	0.089^*^	0.126^**^	0.102^**^	0.134^**^	0.095^**^	−0.068	−0.045	0.084^*^	−0.054	0.018	0.041
Wave 2	0.242^**^	0.208^**^	0.176^**^	0.246^**^	0.162^**^	0.167^**^	0.154^**^	0.208^**^	0.139^**^	0.004	0.004	0.043	−0.035	−0.003	0.023
Wave 3	0.156^**^	0.225^**^	0.228^**^	0.255^**^	0.153^**^	0.127^**^	0.134^**^	0.133^**^	0.129^**^	−0.068	−0.033	0.027	0.049	0.040	0.001
Wave 4	0.177^**^	0.163^**^	0.219^**^	0.221^**^	0.147^**^	0.124^**^	0.142^**^	0.146^**^	0.087^*^	−0.093^**^	−0.062	−0.041	−0.007	0.011	−0.047
Wave 5	0.122^**^	0.187^**^	0.178^**^	0.359^**^	0.183^**^	0.206^**^	0.156^**^	0.131^**^	0.109^**^	−0.076^*^	−0.040	0.036	0.024	0.022	−0.017
Wave 6	0.120^**^	0.183^**^	0.219^**^	0.249^**^	0.316^**^	0.250^**^	0.202^**^	0.246^**^	0.130^**^	−0.060	−0.008	0.015	0.015	0.033	−0.019
Wave 7	0.149^**^	0.178^**^	0.193^**^	0.285^**^	0.263^**^	0.323^**^	0.260^**^	0.280^**^	0.239^**^	−0.047	0.001	0.027	0.015	0.043	−0.019
Wave 8	0.107^**^	0.145^**^	0.179^**^	0.214^**^	0.211^**^	0.240^**^	0.242^**^	0.250^**^	0.209^**^	−0.035	−0.001	0.044	0.050	0.014	0.021
Wave 9	0.093^**^	0.066	0.089^*^	0.159^**^	0.187^**^	0.164^**^	0.130^**^	0.328^**^	0.090^*^	−0.026	−0.007	0.015	0.020	0.015	0.049
Autonomy															
Mean	5.67	5.72	5.75	5.81	5.77	5.64	5.71	5.77	5.74	---details for covariates are in the sample section--					
SD	1.07	0.83	0.74	0.64	0.68	0.68	0.63	0.56	0.60						
Job Satisfaction															
Mean	7.66	7.77	7.80	7.67	7.68	7.74	7.77	7.77	7.86						
SD	1.35	1.25	1.12	1.20	1.09	0.98	1.00	0.88	0.80						

Solo, dummy 0 for solo; sex, dummy 0 for female; income, log annual business income.

* *p* < 0.05;

** *p* < 0.01.

**Table 2:** Fit indices for each model.

Model	*χ*^2^(*df*)	NFI	TLI	CFI	RMSEA	AIC	BIC	Δ*χ*^2^(*df*)
Autonomy								
I	140.76(27)	0.94	0.93	0.95	0.07	176.76	279.62	--
I+L	105.72(25)	0.96	0.95	0.96	0.06	145.72	260.04	35.04(2)^***^
I+L+Q	91.80(22)	0.96	0.95	0.97	0.06	137.80	269.23	13.92(3)^**^
I+L+C	78.68(18)	0.97	0.95	0.97	0.06	132.68	259.96	13.12(4)^**^
Job Satisfaction								
I	105.79(27)	0.95	0.95	0.96	0.06	141.79	226.64	--
I+L	68.14(25)	0.97	0.97	0.98	0.05	108.14	202.42	37.65(2)^***^
I+L+Q	50.64(22)	0.98	0.98	0.99	0.04	91.29	205.06	17.50(3)^***^
I+L+C	37.29(18)	0.98	0.98	0.99	0.03	96.64	218.58	13.35(4)^**^

I, intercept; L, linear; Q, quadratic; C, cubic; NFI, Normed Fit Index; TLI, Tucker Lewis index; CFI, Comparative Fit Index; RMSEA, Root Mean Square Error of Approximation; AIC, Akaike Information Criterion; BIC, Bayesian Information Criterion; Δχ^2^, chi-square difference.

*** *p* < 0.001;

** *p* < 0.01.

To assess the fit of the model, the common fit indices are used, i.e., Normed Fit Index (NFI), Tucker Lewis Index (TLI), Comparative Fit Index (CFI), Root Mean Square Error of Approximation (RMSEA), AIC, and BIC. For NFI, TLI, and CFI, values of 0.90 or higher indicates a good fit of model. RMSEA values closer to 0 represent a good fit. The AIC and BIC compares two different models are estimated and the model with the lowest AIC/BIC are the best fitting model.

**Figure 3:**
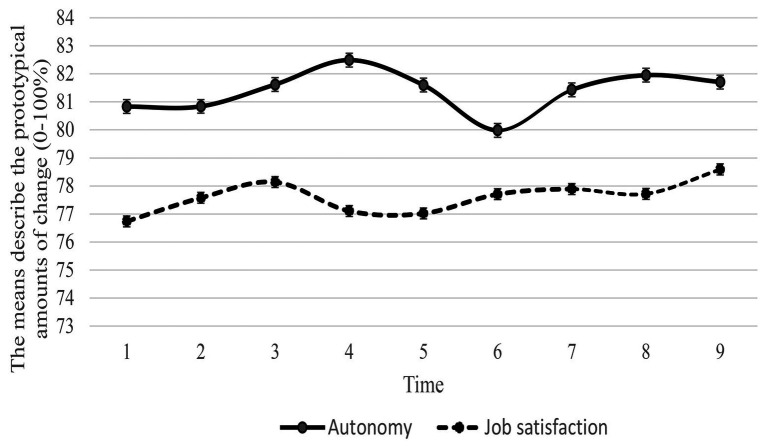
The means describe the prototypical amounts of change in job autonomy and in job satisfaction (independently) across time.

In estimating the trajectory of job autonomy over 9 years, the cubic model provided good fit to the data (*χ*^2^ = 81.26, *df* = 18, *p* < 0.000, NFI = 0.99, TLI = 0.98, CFI = 0.99, RMSEA = 0.03, AIC = 135.26, BIC = 303.99) and was therefore used in all subsequent analysis due to the fit indices. The significant variances indicated that there were significant differences over time (intercept and slope). As can be seen in Figure 3, the means describe the prototypical amounts of change in job autonomy, increasing over the years with a reduced acceleration from wave four to wave six. The job autonomy then accelerated increase from wave six through wave eight, with slightly decline in wave nine.

**Figure 4:**
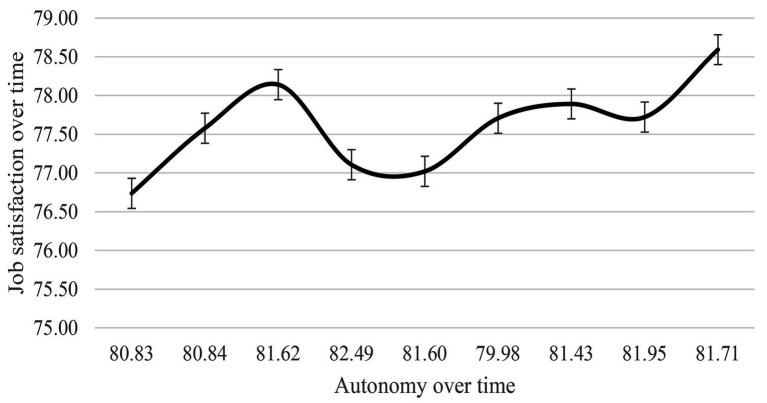
The relationship between job autonomy and job satisfaction (scales are converted into percentage).

In estimating the trajectory of job satisfaction over 9 years, the cubic model provided a good fit to the data (*χ*^2^ = 60.95, *df* = 18, *p* < 0.000, NFI = 0.99, TLI = 0.98, CFI = 0.99, RMSEA = 0.03, AIC = 114.95; BIC = 283.68) and was therefore used in all subsequent analysis due to the fit indices. The significant variances indicated that there were significant differences over time (intercept and slope). As can be seen in Figure 3, the means describe the prototypical amounts of change in job satisfaction, increasing from wave one to three, then declining during wave three to five. The level of job satisfaction increased again during wave five to nine, with slightly drop in wave eight. Figure 4 illustrates the relationship between job autonomy and job satisfaction over time.

### Parallel Process Model

To examine the effects of changing job autonomy on job satisfaction, the parallel process model was used (Figure 2). This approach allows the modeling of two growth trajectories (intercepts and slops) simultaneously. This model adds the distinctive component of directional paths between growth factors, so the study can determine whether the initial status of job autonomy predicts the rate of change of job satisfaction. The fit of the model was good, (*χ*^2^ = 1221.19 (129), *p* < 0.000, NFI = 0.91, TLI = 0.90, CFI = 0.91, RMSEA = 0.05). The results showed, first, that the intercept of job autonomy significantly and positively influenced the intercept of job satisfaction (*β* = 0.61, *p* < 0.001), indicating that higher perceived job autonomy was associated with higher job satisfaction. The intercept of job autonomy significantly and negatively influenced the rate of change in job satisfaction (*β* = −0.32, *p* < 0.001), indicating that initial level of job autonomy predicted growth in job satisfaction. The negative influence means that the increase of job satisfaction was slower among small business owner-managers who started off with a higher level of job autonomy, compared with those who started off with a lower level of job autonomy.

Further, the change rate of job autonomy negatively influenced the initial level of job satisfaction (*β* = −0.25, *p* < 0.001), indicating that small business owner-managers who perceived greater fluctuation of job autonomy reported lower initial level of job satisfaction, compared to those who perceived lesser shifts in job autonomy. However, there was a positive relationship between rate of change of job autonomy and rate of change of job satisfaction (*β* = 0.73, *p* < 0.001), indicating that the change rate of job satisfaction was faster among small business owner-managers who perceived greater fluctuation of job autonomy, comparing to those who perceived lesser shifts in job autonomy.

### Conditional Model

The covariates (gender, age, occupational tenure, business income, industry, number of employees, and solo business owner-managers) were added to the parallel process model (Figure 2). The significant relationship of intercept and slope between job autonomy and job satisfaction remained in the same direction. Only gender had marginal effect on the change rate of job autonomy (*β* = 0.08, *p* < 0.10), indicating that men perceived a greater shift in job autonomy than women. Age also had marginal effect on the initial level of job satisfaction (*β* = 0.09, *p* < 0.10), indicating that older individuals had higher initial levels of job satisfaction.

The results can be summarized as follows. Drawing from the results of unconditional growth models, Hypotheses 1 and 2 were supported, curvilinear growth pattern reflected the change over time for job autonomy and job satisfaction independently. Drawing from results of parallel process model and conditional model, Hypothesis 3a was supported, and the intercept of job autonomy positively predicted intercept of job satisfaction. Hypothesis 3b was not supported, but the reverse effect was found (i.e., intercept of job autonomy negatively predicted growth/slope of job satisfaction). Hypothesis 4a was not supported, but the reverse effect was found (i.e., growth/slope of job autonomy negatively predicted the intercept of job satisfaction). Hypothesis 4b was supported as the growth/slope of job autonomy positively predicted the slope of job satisfaction.

## Discussion

The main motivation of the current study is to understand the role of small business owner-managers’ job autonomy and how it impacts on job satisfaction over time. More specifically, which pattern of these relationship can be best described (e.g., linear pattern, that is job autonomy, decreases over time, which impacts on a reduction of job satisfaction, or non-linear pattern, such as job autonomy, increases to certain periods then decrease, which impacts on a fluctuation of job satisfaction)?

Although literature suggests job autonomy is a prime reason for business start-ups, minimal research has examined to what extent the job autonomy changes over time. Studying job autonomy over time is essential for fully understanding the business ownership context. If job autonomy is a primary motivator, its change over time may impact not only business performance but also job satisfaction.

The current study hypothesized curvilinear growth patterns over time for job autonomy and job satisfaction. The findings illustrate a cubic polynomial and non-monotonic change, this means the cubic spline contains an increase and a decrease (see Figures 3, 4). The findings reflect Van Gelderen (2016) proposal that business ownership does not always result in an ongoing high level of job autonomy and that autonomy is actually dynamics. Business owner-managers may be required to sacrifice their job autonomy in some circumstances, but they are more likely to make an effort to gain it back. This phenomena well reflect the SDT, explaining that an experience of job autonomy is self-determined. Small business owner-managers are motivated primarily by a high level of job autonomy (Gatewood et al., 1995; Carter et al., 2003). The job autonomy can decrease or increase over time (due to various factors such as customers’ requirement or industry regulation); therefore, individuals attempt to maintain the same level of perceived autonomy. That is why the level of job autonomy may be seen as “wiggles” (i.e., undershoots and overshoots). This pattern is also found in the results relating to job satisfaction.

The relationships between job autonomy and job satisfaction can be summarized as follows. Recalling that LGM creates two latent variables (intercept/initial status and slope/growth), the intercept model closely approximates that used in the cross-sectional (one point in time) designs. The current study reflects the cumulative and cross-sectional evidence that the initial status of job autonomy has a positive association with the initial status on job satisfaction. This finding well reflects the DCM, which demonstrates the positive relationship between high job autonomy and job satisfaction (Karasek, 1979; Karasek and Theorell, 1990). Available research shows that high level of job satisfaction among small business owner-managers can be explained by the perceived job autonomy (Hundley, 2001; Benz and Frey, 2004; Stephan, 2018). Additionally, the current study found that small business owner-managers who perceived greater fluctuation in job autonomy over time reported lower initial level of job satisfaction. However, the greater fluctuation in job autonomy stimulates the shift in job satisfaction. This findings echoes the Van Gelderen (2016) proposal that business owner-mangers make an effort to maintain/regain their job autonomy over time. The greater shift in job autonomy may be seen as a challenge stressor (positive stress) that motivates them to negotiate job autonomy with regard to current business context.

### Theoretical and Practical Implications

The current study has several implications for small business theory and practice as well as public policy. First, we advance the current literature by empirically testing the change patterns of job autonomy and its impact on job satisfaction over time. This is important because previous studies have often implied that comparing with regular employees, self-employed individuals experience more autonomy at work and thus they are more satisfied with their job (e.g., Benz and Frey, 2004, 2008a,b; Benzing and Chu, 2009). However, there is still a lack of empirical understanding whether the entrepreneurial autonomy remains unchanged. The qualitative themes emerged from a recent study by Van Gelderen (2016) revealed that the job autonomy among business owners can be reduced due to pressure from key stakeholders. The current study sheds further light by empirically testing and verifying the change patterns of job autonomy and its impact on job satisfaction.

Second, psychology literature, which focused on self-employment or entrepreneurship, has disproportionally focused on the negative aspect of entrepreneurial career, such as entrepreneurial stress (e.g., Buttner, 1992; Cardon and Patel, 2015; Baron et al., 2016; Fernet et al., 2016) and neglecting positive aspect such as job autonomy. While the current study shows that the job autonomy is unstable, the current findings also elaborate that the relationship of job autonomy and job satisfaction over time can swing up and down, approximately a 3-year cycle.

The current empirical findings are also relevant for policymaking and individuals’ entrepreneurial behaviors. When job satisfaction is high, an individual will performance better—this relationship has been described as the “Holy Grail” in industrial and organizational psychology literature (Judge et al., 2001). One way to improve job satisfaction is to reduce work-related stress, such as reducing workload or job demands. For self-employment or entrepreneurial career, workload remains at high level as small business owner-managers are required to work long hours and face many constraints. Additional to workload or job demands monitoring as a risk factor, the level of job autonomy can be tracked as a critical determinant to job satisfaction. A lesson learned from the current study is that job autonomy is unsteady. When the job autonomy is low, additional support may be required. This support can be drawn from personal and institutional resources in order to assist individuals to quickly regain the job autonomy. Family and communities can provide social and emotional support for individuals during the fall of perceived job autonomy. Local business network may be used as a social interactive hub for small business owners to share their experience, strength, and hope with each other. The emotional support can increase individuals’ personal resources (such as self-efficacy, hope, optimism, and resilience), which can be used to prevent any negative emotion and to maintain a self-directed goal in regaining individuals’ autonomy.

Institutional resources can aid small business own-managers’ capability to quickly regain the job autonomy. For example, financial institutions may provide microfinance for improving small business performance. The financial support may help small business owner-managers to gain a confidence and a sense of autonomy over a situation which is restricted by a short-term cash-flow issue. Government-funded small business advisory may provide soft skill trainings such as conflict resolution, negotiation, and mediation tailoring for small business context. Such trainings will equip small business owner-managers with negotiation competency in regaining a sense of job autonomy. Laws and regulations such as weekend penalty rates^8^ can possibly pressure small businesses to involuntarily close on weekends. Regulators may consider to subsidy the additional cost such as tax credit.

As mentioned prior in DCM theory section, job autonomy is a critical determinant of job satisfaction. Work behavior and performance are also determined by the level of job satisfaction (Judge et al., 2001). Therefore, job satisfaction among small business owner-managers is an important aspect. This is because the happy individuals may perform well, and they are likely to maintain in their job. To prevent dissatisfaction, relevant agencies should look into building individual capability in regaining job autonomy as previously mentioned. These resources can act as protective factors in eliminating risk of poor satisfaction over one’s job.

### Limitations and Future Research Directions

While this article has several strengths, there are some limitations that should be considered. The representative panel data, such as HILDA or Global Entrepreneurship Monitor (GEM), can be limited in term of studied variables. Thus, the current results, which are based on the longitudinal panel data, should be complemented by a more in-depth study in which additional variables are considered. For example, start-up motivations may impact on the relationship between self-employment job autonomy and job satisfaction. Necessity and opportunity self-employed may prefer different levels of freedom (Williams, 2007). Further, different motivation typologies may affect the outcome expectation, such as the primary work outcome for self-employed craftsmen is found to be the mastery of the job, not the lure of financial gain, while the opportunistic self-employed individuals may prefer financial success and expansion of the business (Woo et al., 1991).

This study examined the growth pattern of job autonomy only among business owners who remain in a business. For those who quit, other growth patterns may hold. It may be worthwhile for future studies to examine job autonomy patterns among business owner-managers who quit after certain years, comparing the pattern between short-lived business and long-lived business.

Another limitation is the data deriving from the Australian population. The generalizability of the findings toward other nationalities can be questioned. The relationship between job autonomy and well-being can be varied across cultures (Chirkov et al., 2003; Gelderen et al., 2017). The current study scope does not include the cross-cultural or cross-national studies, yet future studies may replicate and examine the relationship between self-employment job autonomy and its impact from other panel data such as British Household Panel Survey or German Socio-Economic Panel. The challenge is that these panels should at least capture the similar variables for a comparison.

## Conclusion

The current study examined the relationship between job autonomy and job satisfaction among small business owner-managers. As described in the introduction, the topic of entrepreneurial job autonomy and particularly in a longitudinal context are under-researched. The current study contributes to the current knowledge by empirically and longitudinally examining this relationship, which was previously suggested by Van Gelderen’s qualitative work. The current study confirmed the proposed hypotheses that job autonomy among small business owner-managers fluctuates over time, and the greater fluctuation individuals perceive, they feel less satisfied job.

## Data Availability Statement

The data analyzed in this study was obtained from National Centre for Longitudinal Data Dataverse. Requests to access these datasets should be directed to Institute for Quantitative Social Science, melb-inst@unimelb.edu.au.

## Ethics Statement

Ethical review and approval was not required for the study on human participants in accordance with the local legislation and institutional requirements. Written informed consent from the [patients/ participants OR patients/participants legal guardian/next of kin] was not required to participate in this study in accordance with the national legislation and the institutional requirements.

## Author Contributions

SS developed the paper ideas, theoretical framework, and mentoring data analysis. PO’C developed the introduction, oversaw the overall structure and flow. SS and RK performed analysis. SS and PJ contributed to the implication. All authors contributed to the article and approved the submitted version.

### Conflict of Interest

The authors declare that the research was conducted in the absence of any commercial or financial relationships that could be construed as a potential conflict of interest.

## Footnotes

^1^Drawing from a commonly used behavioral definition, entrepreneurship as independent ownership, active management, and/or expressed intention to do so (Stewart and Roth, 2001). In the current study, entrepreneurs are defined as individuals who control an ownership of enterprise, and the term owner-managers are used interchangeably.

^2^Authors’ calculations based on Table 8.32 in Summerfield et al., 2016.

^3^The current study included all active self-employed individuals who are also over 50 years old, reflecting the senior entrepreneur classification. For more information about senior entrepreneurship, please see Perenyi et al. (2018).

^4^For other industry sector such as manufacturing, transport, etc. the figures were less than 9% in each category.

^5^(1) I have many freedom to decide how I do my own work, (2) I have a lot of say about what happens on my job, (3) I have a lot of freedom to decide when I do my work, (4) I have a lot of choice in deciding what I do at work, (5) My working times can be flexible, and (6) I can decide when to take a break.

^6^A single latent variable (job autonomy) is measured by several observed variables (six items).

^7^A few studies suggested the U-shaped relationship between age and satisfaction (Blanchflower and Oswald, 2000; Sloane and Williams, 2000). This article also tested Age and Age2, but there was non-significant relationship.

^8^In Australia, employers are to pay at least 150% (time and a half) of the normal base wage for work performed on a Saturday and 200% (double time) for employees who perform work on a Sunday.
